# Clinical diagnostic performance of droplet digital PCR for pathogen detection in patients with *Escherichia coli* bloodstream infection: a prospective observational study

**DOI:** 10.1186/s12879-024-10396-y

**Published:** 2025-01-06

**Authors:** Hiroki Kitagawa, Masato Kojima, Kayoko Tadera, Shuta Kogasaki, Keitaro Omori, Toshihito Nomura, Norifumi Shigemoto, Eiso Hiyama, Hiroki Ohge

**Affiliations:** 1 Department of Infectious Diseases, Hiroshima University Hospital, 1-2-3 Kasumi, Minami-ku, Hiroshima, 734-8551 Japan; 2 Department of Surgery, Graduate School of Biomedical and Health Sciences, Hiroshima University, 1-2-3 Kasumi, Minami-ku, Hiroshima, 734-8551 Japan; 3 Department of Pediatric Surgery, Hiroshima University Hospital, Hiroshima, 734-8551 Japan; 4 Department of Biomedical Science, Natural Science Center for Basic Research and Development, Hiroshima University, Hiroshima, Japan; 5 Section of Clinical Laboratory, Division of Clinical Support, Hiroshima University Hospital, Hiroshima, 734-8551 Japan; 6 Division of Laboratory Medicine, Hiroshima University Hospital, Hiroshima, 734-8551 Japan; 7 School of Medicine, Hiroshima University, Hiroshima, 734-8551 Japan; 8 Translational Research Center, Hiroshima University, Hiroshima, 734-8551 Japan

**Keywords:** Droplet digital PCR, Bloodstream infection, Blood culture, Sepsis, Time-to-positivity, Mortality

## Abstract

**Background:**

Droplet digital PCR (ddPCR) is a highly sensitive tool for detecting bacterial DNA in bacterial bloodstream infections (BSI). This study aimed to examine the sensitivity and specificity of ddPCR and the association between bacterial DNA load in whole blood and the time-to-positivity (TTP) of blood culture (BC) in patients with *Escherichia coli* BSI.

**Methods:**

This prospective study enrolled patients with *E. coli* BSI confirmed via BC at the Hiroshima University Hospital from June 2023 to August 2024. The *E. coli* DNA load in whole blood, which was simultaneously obtained from two BC sets, was measured using ddPCR with *E. coli* specific primer and probe. Whole blood samples from 50 patients with BC positive for pathogens other than *E. coli* (*n* = 25) and BC negative (*n* = 25) were also evaluated using ddPCR.

**Results:**

A total of 131 patient samples were analyzed in this study. Of the 81 patients with *E. coli* BSI, ddPCR detected *E. coli* DNA in 67 (82.7%). The results of ddPCR for *E. coli* had a sensitivity of 82.7% (95% CI: 73.1–89.4%), specificity 100% (95% CI: 93.0–100%). Patients with positive ddPCR results had significantly shorter TTP than those with negative results (median, 8.8 h vs. 10.7 h, *p* < 0.001). The positivity rate for both BC sets was significantly higher in patients with positive ddPCR results than in those with negative results (89.6% vs. 35.1%, *p* < 0.001). Among ddPCR-positive patients, septic shock was significantly associated with intestinal perforation, higher *E. coli* DNA load, higher 28-d mortality, shorter TTP, and higher positivity rate for four bottles of BC than those without septic shock. The *E. coli* DNA load in whole blood negatively correlated with TTP (*p* < 0.001, R^2^ = 0.38).

**Conclusion:**

The *E. coli* DNA load in whole blood is inversely correlated with TTP. Notably, a higher *E. coli* DNA load is associated with septic shock.

## Background

Bacterial bloodstream infections (BSI) and the associated sepsis/septic shock are among the leading causes of mortality in critically ill patients. Rapid diagnosis and early administration of appropriate antimicrobials are crucial for improving the outcomes and reducing the mortality rate in the patients with BSI [1, 2]. Currently, blood culture (BC) is the conventional gold standard for detecting pathogenic microorganisms and antimicrobial susceptibility testing for the diagnosis of BSI [3]. However, BC is limited by low sensitivity and long turnaround time [4].

Molecular technologies, such as real-time quantitative polymerase chain reaction (RT-qPCR) and next-generation sequencing, have been used to directly detect pathogens and antimicrobial resistance genes in blood samples without prior incubation to improve BSI diagnosis [5]. However, some of these techniques have limited sensitivity or specificity and high operational costs, so are unsuitable for use in clinical practice [5]. In recent years, droplet digital PCR (ddPCR), the third generation of PCR, has emerged as a new molecular method for the diagnosis of BSI [6–12]. DdPCR is more sensitive than RT-qPCR and quantifies nucleic acids without generating a calibration curve [6–12]. Hu et al. [8] compared ddPCR with metagenomic next-generation sequencing (mNGS) in critically ill patients and found that the target pathogen range of the ddPCR assay had a higher detection rate of blood pathogens than that of the mNGS assay, whereas the range of pathogens detected by plasma DNA mNGS was wider than that detected by ddPCR. However, the accuracy of pathogen detection using ddPCR and the association between ddPCR and BC results have not yet been fully investigated.

In this study, we aimed to evaluate the sensitivity and specificity of ddPCR in diagnosing BSI caused by *Escherichia coli*, which is one of the most common pathogens responsible for BSI, in a real-world clinical setting. We also examined the association between bacterial DNA load in whole blood and the time-to-positivity (TTP) of BC in patients with *E. coli* BSI. In addition, we investigated the association of bacterial DNA load with disease severity.

## Materials and methods

### Spiked blood experiment

*E. coli* ATCC25922 was plated on 5% sheep blood agar plates (Eiken Chemical Co., Ltd, Tokyo, Japan) and incubated for 24 h under aerobic conditions at 35 ℃. The initial bacterial suspension was adjusted to 0.5 McFarland in 0.9% sterile saline. The concentrations of the bacterial suspension were determined using the plate count method and serial dilution. This suspension was serially diluted (1:10) in 0.9% sterile saline, and 50 µL of the diluted bacterial suspension were plated on 5% sheep blood agar plates under the same conditions as initial bacteria. Colonies were counted and CFU/mL of the initial suspension was calculated. Thereafter, the initial suspension was serially diluted (1:10) in human blood of a healthy volunteer and 1 mL aliquots of human blood were spiked with four different concentrations of *E. coli* ranging from 10^4^ to 10^1^ CFU/mL. Subsequently, 400 µL of the spiked blood samples were used for DNA extraction using the automatic extraction system magLEAD^®^ with magLEAD^®^ Dx SV reagent (Precision System Science Co., Ltd., Chiba, Japan), following the manufacturer’s instructions. The final eluate (50 µL) was stored at − 80 °C until further use for ddPCR. The bacterial concentrations of the spiked samples at each concentration were also determined by culture. The spiked samples were serially diluted (1:10) using human blood, and 50 µL of the diluted spiked sample was plated on 5% sheep blood agar plates under the same conditions as the initial bacteria. Colonies were counted and the number of CFU/mL in the initial spiked samples was calculated. These experiments were performed in duplicate and the mean of the two results was used in the analysis.

### ddPCR

The primers and probes designed to target the *E. coli* specific gene SWG-9 (Integrated DNA Technologies, Coralville, IA, USA) were used to detect *E. coli*, as described previously (Forward primer: 5’TCACGCCGTATGTTATTG-3’, Reverse primer: 5’GTCGGTAATCACCATTCC-3’, and Probe: 5’FAMTGCCAGTTCAGTTCGTTGTTCAC-BHQ1-3’) [13]. All assays were performed on a QX100 droplet digital PCR system (Bio-Rad Laboratories, Inc., Pleasanton, CA, USA), according to the manufacturer’s instructions [14]. Each ddPCR reaction included 22 µL of reaction mixture, which comprised 5 µL of template DNA, 10 µL of 2× ddPCR Supermix for probes (no dUPT) (Bio-Rad Laboratories, Inc.), 0.9 µL each of the forward and reverse primers (10 µM), 0.25 µL of the probe (10 µM), and 4.95 µL of DNase-free water. Thereafter, the reaction mixture was placed in an automated droplet generator (Bio-Rad Laboratories, Inc.) to generate droplets. The generated droplet emulsions were transferred to a new 96-well PCR plate and amplified on a ProFlex PCR System (Thermo Fisher, Waltham, MA, USA) using the following protocol: 95 °C for 10 min, 40 cycles at 94 °C for 30 s and 57 °C for 1 min, and a final cycle at 98 °C for 10 min. After gene amplification, the plates were transferred to a QX100 droplet reader (Bio-Rad Laboratories, Inc.). The Bio-Rad QuantaSoft software (Bio-Rad Laboratories, Inc.) was used to determine the number of droplets containing the target DNA molecule and to calculate the concentration of target DNA molecules in the reaction mixture copies/µL.

The number of target DNA molecules in the whole blood samples was calculated as copies/mL. To estimate the limit of detection, serial dilutions (1:10) of DNA template of *E. coli* ATCC25922 extracted from spiked whole blood described above in 0.9% sterile saline and DNA extracted from whole blood of a healthy volunteer with no spiked *E. coli* as a negative control, were evaluated using ddPCR. In this study, the limit of detection of this assay was determined as three droplets containing the target DNA molecule, which was calculated to be approximately 75 copies/mL in whole blood.

### Study design and patients

This single-center prospective observational study was conducted at Hiroshima University Hospital, a tertiary care hospital in Japan, from June 2023 to August 2024. The inclusion criteria for patient enrollment were as follows: (1) ≥ 18 years of age, (2) patients from whom two sets of BC were obtained simultaneously, along with whole blood samples, and (3) BC positive for *E. coli*. The exclusion criteria were as follows: (1) patients who were treated with antimicrobial agents within 1 week prior to collection of BC, (2) presence of polymicrobial bacteremia, (3) patients whose residual volume of whole blood sample was less than 400 µL, and (4) patients who died on the same day of BC sampling (Table 1). Polymicrobial bacteremia was defined as the isolation of more than one microorganism from a BC. Residual whole blood samples from 50 patients with BC positive for pathogens other than *E. coli* (*n* = 25) as well as those with BC negative (*n* = 25) were evaluated using ddPCR with *E. coli* specific primer and probe as negative control. The pathogens other than *E. coli* included *Staphylococcus aureus* (*n* = 5), *Staphylococcus epidermidis* (*n* = 2), *Enterococcus faecalis* (*n* = 3), *Enterococcus faecium* (*n* = 1), *Streptococcus pneumoniae* (*n* = 1), *Klebsiella pneumoniae* (*n* = 5), *Klebsiella aerogenes* (*n* = 2), *Enterobacter cloacae complex* (*n* = 2), *Pseudomonas aeruginosa* (*n* = 2), *Acinetobacter baumannii* (*n* = 1), *Serratia marcescens* (*n* = 1), *Stenotrophomonas maltophilia* (*n* = 1), *Proteus mirabilis* (*n* = 1), and *Candida albicans* (*n* = 1). These patients were (1) ≥ 18 years of age, (2) those from whom two sets of BC were obtained simultaneously, along with whole blood samples, (3) no polymicrobial bacteremia (4) no administration of an antimicrobial agent 1 week prior to collection of BC.

**Table 1:** Inclusion and exclusion criteria

Category	Criteria	Number excluded
Inclusion criteria (*N* = 113)	1. ≥ 18 years of age	
	2. Patients from whom two sets of blood cultures were obtained simultaneously, along with whole blood samples	
	3. Blood culture positive for *Escherichia coli*	
Exclusion criteria (*N* = 32)	1. Patients who were treated with antimicrobial agents within 1 week prior to collection of blood cultures	16
	2. Presence of polymicrobial bacteremia	13
	3. Patients whose residual volume of whole blood sample was less than 400 µL	1
	4. Patients who died on the same day of blood culture sampling	2

Total number of patients included in the analysis: 113 − 32 = 81

### Blood culture and pathogen identification

Two sets of BC (four bottles in total) were drawn from each patient, according to routine clinical procedures. Whole blood (8–10 mL) was inoculated into each BC bottle (BacT/ALERT FA and FN Plus bottle; bioMérieux, Marcy l’Étoile, France) and incubated at 37 °C in a BacT/ALERT^®^ Virtuo System (bioMérieux). The inoculated volume in each BC bottle was confirmed using the BacT/ALERT^®^ Virtuo System (bioMérieux). The BC were incubated for a maximum of 7 d. For each patient, only the TTP of the first positive BC bottle was used for analysis.

Once a positive signal was reported by the system, Gram staining was performed, followed by subculture, as previously described [15]. The isolates from BC were identified via matrix-assisted laser desorption/ionization time-of-flight mass spectrometry using a MALDI Biotyper Sirius system (Bruker Daltonik GmbH, Bremen, Germany), as described previously [15]. Extended-spectrum β-lactamase production was screened and confirmed using disk diffusion methods, as described in Clinical and Laboratory Standards Institute M100-Ed33 [16].

### Whole blood DNA extraction and ddPCR testing

#### Sample collection and DNA extraction

As *E. coli* BSI were not known at the time of BC submission, DNA was extracted and stored from all whole blood samples collected at the same time as the BC of patients with suspected BSI during the study period. Whole blood samples (1–1.5 mL) collected using ethylenediaminetetraacetate anticoagulant tubes were used for ddPCR detection. These blood samples were residues of samples obtained in clinical practice. DNA was extracted on the same day or the day after the whole blood sampling. Whole blood samples were stored at room temperature if DNA was extracted on the day of collection. If DNA was extracted at a later date, it was stored in a refrigerator at 4 °C. DNA was extracted from 400 µL of whole blood sample using the automatic extraction system magLEAD^®^ with MagDEA^®^ Dx SV reagents (Precision System Science Co.), following the manufacturer’s instructions. The final eluate (50 µL) was stored at − 80 °C until further use in ddPCR.

### Data collection

All clinical data, including demographic data, primary site of BSI, vital signs, the presence of septic shock at the time of BC sample collection, BC results, laboratory data, and 28-d mortality rate, were collected from the hospital’s electronic medical system. Septic shock was defined as previously described [17].

### Ethics

The study protocol was approved by the Ethical Committee for Epidemiology of Hiroshima University (E-2133). The requirement for written informed consent was waived by the Ethical Committee for Epidemiology of Hiroshima University because of the noninvasive study design as only residual whole blood samples were used.

### Statistical analysis

Categorical data are expressed as frequencies and proportions (%), and continuous variables are expressed as medians and interquartile ranges (IQR). All categorical variables were compared using Fisher’s exact test or χ2 test, as appropriate, and continuous variables were compared using the Mann–Whitney U test. The sensitivity, specificity, positive predicted value (PPV), and negative predicted value (NPV) were calculated using the Wilson’s score method, along with the 2-sided 95% confidence interval (CI). All *p* values were two-sided, and statistical significance was set at *p* < 0.05. Pearson correlation coefficient was used to compare the *E. coli* concentration evaluated using bacterial culture (log_10_ [CFU/mL]) with the *E. coli* DNA load in whole blood (log_10_ [copies/mL]) obtained from the ddPCR assay performed for the spiked blood experiment. Further, the relationship between the *E. coli* DNA load in whole blood (log_10_ [copies/mL]) and the TTP of BC (hours) were assessed using the Pearson correlation coefficient. Data analysis was performed using the JMP Pro software (version 16.0; SAS Institute Inc., Cary, NC, USA).

## Results

### Spiked blood experiment

**Fig. 1:**
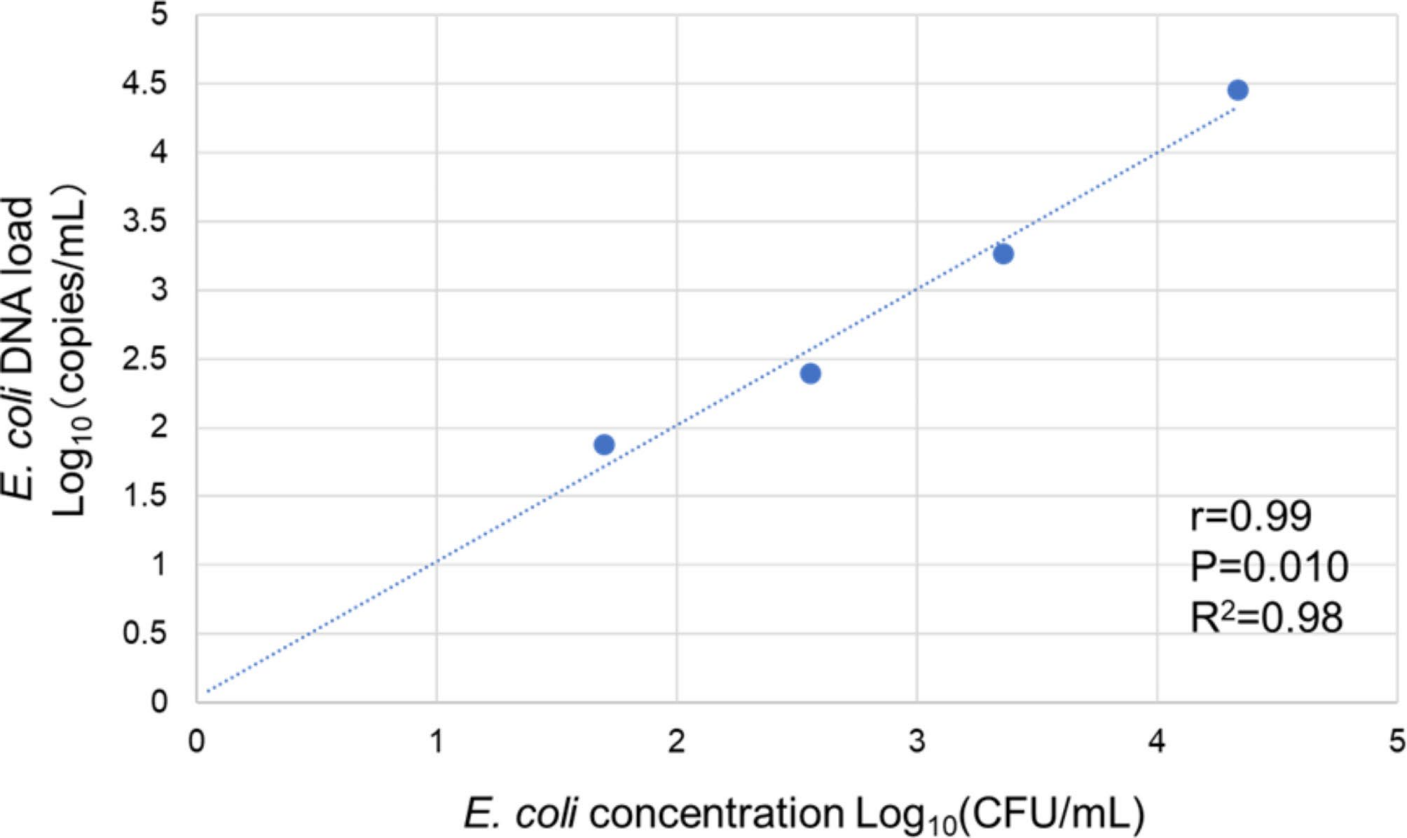
Correlation between the *E. coli* concentrations evaluated by the bacterial culture (log_10_(CFU/mL)) and the number of gene copies/mL detected by ddPCR assay for detecting *E. coli* (log_10_(copies/mL))

A significant correlation (*r* = 0.99, 95% CI: 0.58–1.00, *p* = 0.010, R^2^ = 0.98) was observed between the *E. coli* concentration determined by bacterial culture and the *E. coli* DNA load detected by ddPCR (Fig. 1).

### Clinical study

Eighty-one patients with *E. coli* BSI confirmed by BC were included in this study. Using ddPCR, *E. coli* DNA was detected in 82.7% (67 of 81 patients) of the whole blood samples, but it was not detected in any of the 50 negative control samples (Table 2). The results of ddPCR for *E. coli* exhibited a sensitivity of 82.7% (95% CI: 73.1–89.4%), specificity of 100% (95% CI: 93.0–100%), PPV of 100% (95% CI: 94.6–100%), and NPV of 78.1% (95% CI: 66.6–86.5%).

**Table 2:** The results of blood culture and ddPCR for *Escherichia coli*

	Blood culture for E. coli		Total
	Positive	Negative*	
ddPCR for *E. coli*			
Positive	67	0	67
Negative	14	50	64
Total	81	50	131

* The patients with negative blood culture for *E. coli* included 25 patients with blood culture positive for pathogens other than *E. coli* and 25 patients with negative blood culture

The clinical characteristics and details of the BC results of the patients who tested positive or negative by ddPCR are shown in Table 3. Patients with positive ddPCR results had significantly shorter TTP than those with negative ddPCR results (median TTP, 8.8 h vs. 10.7 h, *p* < 0.001). The rate of positive results for both sets of BC in patients with positive ddPCR results was significantly higher than in patients with negative ddPCR results (89.6% vs. 35.1%, *p* < 0.001).

**Table 3:** Clinical characteristics and detailed information of the blood culture results of patients who had positive or negative ddPCR results

	All patients (*n* = 81)	Patients with positive ddPCR results (*n* = 67)	Patients with negative ddPCR results (*n* = 14)	*P* value
Age (years), median (IQR)	74 (66–82)	75 (60–82)	70 (56–79)	0.14
Male, *n* (%)	46 (56.8)	38 (56.7)	8 (57.1)	0.97
Primary site of BSI				
Abdominal infection, *n* (%)	42 (51.9)	38 (56.7)	4 (28.6)	0.078
Bile duct infection, *n* (%)	34 (42.0)	30 (44.8)	4 (28.6)	0.37
Intestinal perforation, *n* (%)	5 (6.2)	5 (7.5)	0 (0)	0.58
Urinary tract infection, *n* (%)	33(40.1)	24 (35.8)	8 (57.1)	0.14
Others, *n* (%)	6 (7.4)	5 (7.5)	2 (14.3)	0.60
BSI caused by ESBL-producing *E. coli*, *n* (%)	19 (23.5)	15 (22.4)	4 (28.6)	0.73
Septic shock, *n* (%)	17 (21.0)	17 (25.4)	0(0)	0.034
28-day mortality, *n* (%)	8 (9.9)	8 (11.9)	0(0)	0.34
Blood culture				
TTP (h), median (IQR)	9.1 (8.4–10.5)	8.8 (8.3–10.1)	10.7 (9.9–12.5)	< 0.001
Positive for two sets of blood culture, *n* (%)	65 (80.2)	60 (89.6)	5 (35.1)	< 0.001
No. of positive bottle of two sets of blood culture				
4 bottles, *n* (%)	50 (61.7)	48 (71.6)	2 (14.3)	< 0.001
3 bottles, *n* (%)	10 (12.5)	7 (10.5)	3 (21.4)	0.37
2 bottles, *n* (%)	10 (12.5)	10 (14.3)	0 (0)	0.20
2 bottles of different sets, *n* (%)	5 (6.3)	5 (7.5)	0 (0)	0.58
1 bottle, *n* (%)	11 (13.8)	2 (3.0)	9 (64.3)	< 0.001

ESBL, extended-spectrum β-lactamase IQR, interquartile range; TTP, Time-to-positivity; ESBL, Extended-Spectrum β-Lactamase

**Fig. 2:**
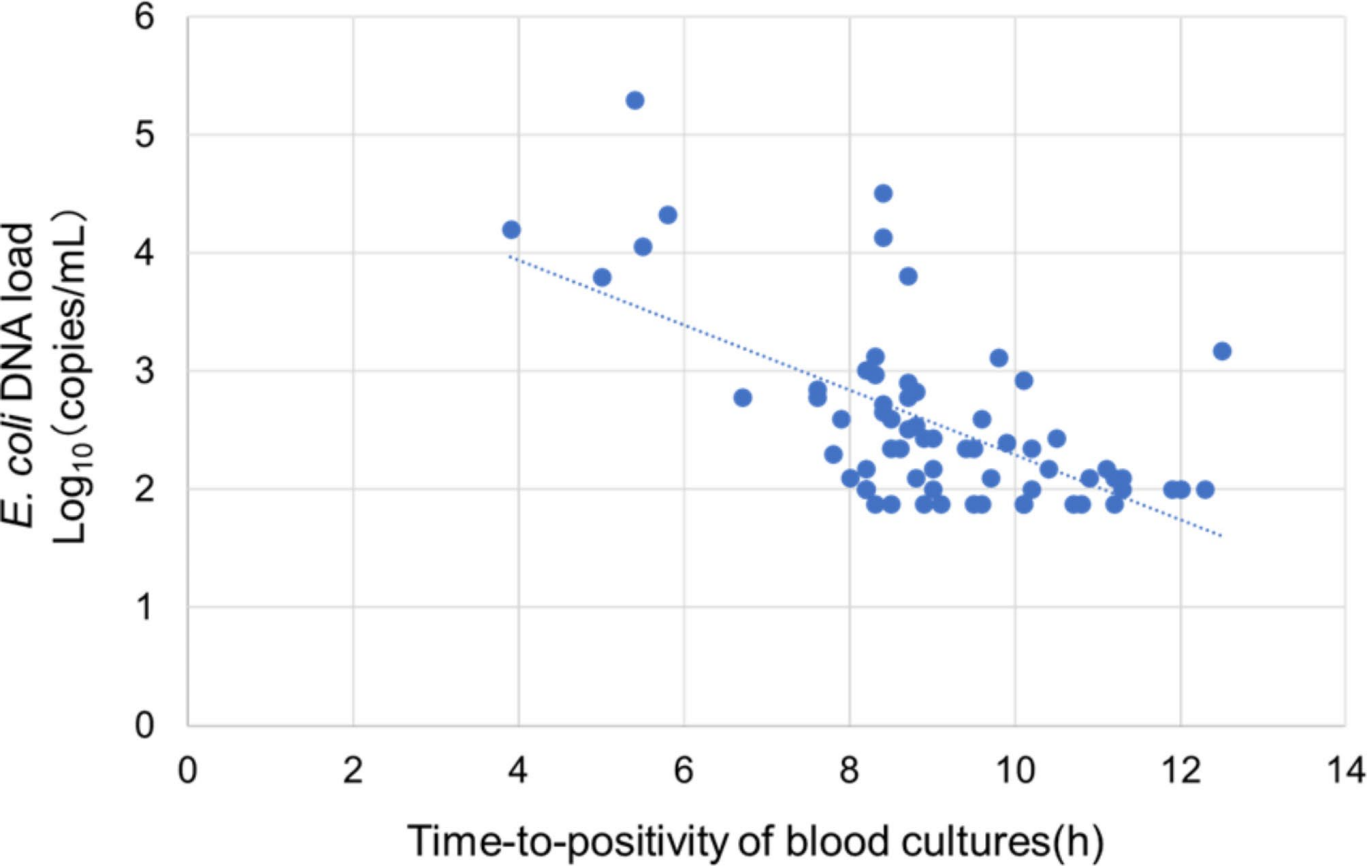
Correlation between *E. coli* DNA load in whole blood and time-to-positivity of blood cultures

The clinical characteristics and details of the BC results of ddPCR-positive patients with and without septic shock are listed in Table 4. The median *E. coli* DNA load in the whole blood was 362 copies/mL (IQR: 200–834 copies/mL). Patients with septic shock had intestinal perforation (29.4% vs. 0%, *p* < 0.001), higher *E. coli* DNA load in whole blood (median *E. coli* DNA load, 6500 copies/mL vs. 312 copies/mL, *p* < 0.001), higher 28-d mortality (47.1% vs. 0%, *p* < 0.001), shorter TTP (median TTP, 8.2 h vs. 9.3 h, *p* < 0.001), and higher positivity rate for the four bottles of BC (100% vs. 62.0%, *p* = 0.016) compared with patients without septic shock. In the 67 patients with positive ddPCR results, the *E. coli* DNA load in whole blood and TTP were significantly negatively correlated (*r* = − 0.62, 95% CI: −0.74 to − 0.44, *p* < 0.001, R^2^ = 0.38; Fig. 2).

**Table 4:** Clinical characteristics and detailed information of the blood culture results of ddPCR-positive patients with and without septic shock

	All patients (*n* = 67)	Patients with septic shock (*n* = 17)	Patients without septic shock (*n* = 50)	*P* value
Age (years), median (IQR)	75 (60–82)	77 (73–84)	75 (68–82)	0.77
Male, *n* (%)	38 (56.7)	10 (58.8)	28 (56.0)	0.84
Primary site of BSI				
Abdominal infection, *n* (%)	38 (56.7)	8 (47.1)	30 (60.0)	0.35
Bile duct infection, *n* (%)	30 (44.8)	3 (17.7)	27 (52.3)	0.011
Intestinal perforation, *n* (%)	5 (7.5)	5 (29.4)	0 (0)	< 0.001
Urinary tract infection, *n* (%)	24 (35.8)	7 (41.2)	17 (34.0)	0.59
Others, *n* (%)	5 (7.5)	2 (11.8)	3 (6.0)	0.59
BSI caused by ESBL-producing *E. coli*, *n* (%)	15 (22.4)	6 (35.3)	9 (18.0)	0.14
*E. coli* DNA load in whole blood (copies/mL), median (IQR)	362 (200–834)	6500 (1106–17512)	312 (138–506)	< 0.001
28-day mortality, *n* (%)	8 (11.9)	8 (47.1)	0(0)	< 0.001
Blood culture				
TTP (h), median (IQR)	8.8 (8.3–10.1)	8.2 (5.7–8.7)	9.3 (8.5–10.3)	< 0.001
Positive for two sets of blood culture, *n* (%)	60 (89.6)	17(100)	43 (86.0)	0.18
No. of positive bottle of two sets of blood culture				
4 bottles, *n* (%)	48 (71.6)	17(100)	31 (62.0)	0.016
3 bottles, *n* (%)	7 (10.5)	0 (0)	7 (14.0)	0.18
2 bottles, *n* (%)	10 (14.3)	0 (0)	10 (20.0)	0.055
2 bottles of different sets, *n* (%)	5 (7.5)	0 (0)	5 (10.0)	0.56
1 bottle, *n* (%)	2 (3.0)	0 (0)	2 (4.0)	1.0

ESBL, extended-spectrum β-lactamase; IQR, interquartile range; TTP, time-to-positivity

## Discussion

To our knowledge, our study includes the largest cohort of patients with *E. coli* BSI for evaluating the accuracy of pathogen detection using ddPCR and the association between ddPCR and BC results. In this study, ddPCR revealed that 82.7% of the whole blood samples from patients with *E. coli* BSI diagnosed using BC were positive. The spiked blood control experiments in this study revealed a significant correlation between the *E. coli* concentrations evaluated by the bacterial culture and the *E. coli* DNA load detected by ddPCR, which is consistent with the results of a previous study [18]. In addition, this study showed a correlation between *E. coli* DNA load in whole blood and a short TTP. Negative ddPCR results were associated with longer TTP and positivity for only one of the two sets of BC. The bacterial count in the whole blood of patients with negative ddPCR results may be too low to be detected by ddPCR but can be detected by BC, which uses a larger sample volume and increases the number of bacteria via incubation. Ziegler et al. [7] reported that 64% (9/14) of whole blood samples from patients with *E. coli* BSI diagnosed via BC were positive based on ddPCR using *E. coli* specific primers and probe. These results are consistent with those of this study. In contrast, in the studies by Lin et al. [10] and Li et al. [11], DNA was extracted from the plasma and multiplex ddPCR (Pilot Gene Technologies. Hangzhou, China) was performed, and *E. coli* DNA was detected via ddPCR in all samples of patients with *E. coli* BSI diagnosed via BC. In addition, approximately half of patients with blood samples that were ddPCR-positive for *E. coli* DNA tested negative in the BC [10, 11]. The previous studies did not investigate the TTP of BC or the number of positive BC bottles [7, 10, 11]. Further studies are needed to investigate the association between ddPCR sensitivity/specificity and BC results.

The use of ddPCR, which can rapidly identify pathogenic microorganisms and antimicrobial resistance genes in blood samples, has great potential as a new diagnostic method for BSI. Compared with conventional molecular technologies, such as RT-qPCR and NGS, ddPCR has the advantage of higher sensitivity and specificity for identifying target genes in clinical samples and dynamic monitoring of pathogen load [5]. However, compared with BC, ddPCR test has the limitations of high operational costs and not being able to perform antimicrobial susceptibility testing of pathogens. Further research is needed to determine which specimens and pathogens should be tested by ddPCR for early diagnosis of infections and improved patient outcomes.

In this study, all patients with septic shock caused by *E. coli* tested positive by ddPCR. Patients with septic shock had a higher *E. coli* DNA load in whole blood, higher 28-day mortality, shorter TTP, and higher positivity rate for the four bottles of BC compared with patients without septic shock. Previous studies have also shown an association between high initial pathogen DNA load in the blood and sepsis/septic shock or mortality [6, 19–22]. Other studies have also shown that a short TTP is associated with mortality among patients with BSI caused by several pathogens [23, 24]. This can be attributed to an almost perfect correlation between the number of CFU/mL of the bacterial culture and the number of gene copies/mL detected by ddPCR, which was found in this study as well as in a previous study [18].

However, some studies reported contradictory results regarding the association between the initial pathogen DNA load and mortality [9, 12]. Ziegler et al. [9] and Zhao et al. [12] did not find a significant association between the initial pathogen load and mortality. The change in the pathogen DNA load in the blood has been proposed as a potential surrogate prognostic marker in BSI assessments [6, 9, 12, 20]. In studies using multiplex ddPCR, Shao et al. [9] and Zhao et al. [12] reported that an increased pathogen DNA load in the blood compared with the baseline load was associated with poor prognosis. There was an overall decline of pathogen DNA load in blood of patients who survived, whereas the load increased in non-survivors [9, 12]. Chuang et al. [20] evaluated the association between bacterial DNA load in blood and mortality in patients with *Acinetobacter baumannii* bacteremia using RT-qPCR assay. High maximum bacterial DNA load and an increasing bacterial DNA load from day 0 to day 3 were independent predictors of mortality in the multivariable analysis. However, in this study, changes in the pathogenic DNA load in the blood over time were not investigated. Further studies with larger sample sizes are needed to determine the association of changes in pathogen DNA load in the blood with disease severity and mortality.

Our study has several limitations. Only patients with *E. coli* BSI were included in this study. The applicability of the results of this study to patients with BSI caused by other species, particularly gram-positive bacteria, has not been studied. Therefore, it is unclear whether the results of this study can be generalized to BSI caused by other bacterial species. In real-world settings, approximately 10% of BC are culture positive. Therefore, if ddPCR is introduced into clinical practice, it must be performed on a large number of negative BC, increasing the microbiology laboratory costs. However, this approach may be beneficial if the clinical microbiology laboratory provides the ddPCR test in selected patients, such as patients with septic shock, as soon as a blood sample is drawn from the patient. In this study, all patients who met the inclusion and exclusion criteria had relatively short TTP of BC, ranging from 3.9 to 13.8 h. In addition, only the TTP of the first positive BC bottle was used for analysis. Therefore, whether ddPCR can identify *E. coli* DNA in the whole blood of patients with BSI and a longer TTP was not studied here. Previous studies have shown an association between changes in pathogenic DNA load in the blood and mortality [6, 9, 12, 20]. However, only the initial pathogenic DNA load was investigated in this study. Furthermore, antimicrobial resistance genes were not investigated. Because this was an observational study without interventional treatment, the clinical benefits of ddPCR could not be accurately evaluated.

In conclusion, this study showed that 82.7% of whole blood samples from patients with *E. coli* BSI diagnosed via BC were ddPCR-positive. Patients with septic shock had significantly higher levels of *E. coli* DNA in the whole blood than those without septic shock. In patients with septic shock, ddPCR may diagnose bacteremia earlier than BC and with accuracy comparable to BC. Further studies are needed to evaluate the accuracy of ddPCR for the diagnosis of patients with BSI caused by other organisms and the association of pathogen DNA load with disease severity and mortality.

## Data Availability

The datasets used and analyzed during the current study are available from the corresponding author on reasonable request.

## Abbreviations

BSI: Bacterial bloodstream infection
BC: Blood culture
CI: Confidence interval
ddPCR: Droplet digital PCR
IQR: Interquartile range
mNGS: Metagenomic next-generation sequencing
NPV: Negative predicted value
PPV: Positive predicted value
RT-qPCR: Real-time quantitative PCR
TTP: Time-to-positivity
